# Variables for habitat and vertebrate hosts of *Ixodes scapularis* are the best ecological predictors of the spatial spread of Lyme disease in the United States (2010–2019)

**DOI:** 10.1186/s13071-025-07047-9

**Published:** 2025-10-14

**Authors:** Patrick H. Kelly, Sarah Willis, Alexander Davidson, James H. Stark, Jennifer C. Moїsi, Agustín Estrada-Peña

**Affiliations:** 1 U.S. Medical Affairs, Vaccines, U.S. Commercial Division, Pfizer, Collegeville, PA USA; 2 Pfizer Research & Development, Global Vaccines Medical Affairs, Cambridge, MA USA; 3 Pfizer Research & Development, Global Vaccines Medical Affairs, Paris, France; 4 Senior External Consultant, Ministry of Health, Government of Spain, Madrid, Spain

**Keywords:** Tick, Ixodid, Ixodidae, Tick-borne, Climate, landscape, hosts, Epidemiology, Modeling

## Abstract

**Background:**

Lyme disease (LD) is a major public health concern in North America. The incidence of LD has increased in part due to the rapid expansion of *Ixodes scapularis* infected with *Borrelia burgdorferi* sensu lato (*Bb*), the causative agent of LD. Understanding how environmental factors contribute to the spread of LD in humans remains a major challenge.

**Methods:**

We aimed to measure the environmental associations and spatial dynamics of LD incidence across United States counties between 2010 and 2019 via a machine-learning (ML)-based model. We used LD incidence data in 1322 counties in 24 US states from the Centers for Disease Control and Prevention (CDC), categorized by four incidence classes (0–1, > 1 to 10, > 10 to 100, > 100 cases/100,000 population). Explanatory variables of climate, habitat, land cover, *I. scapularis* presence, and distribution of tick hosts and *Bb* reservoirs were used to train the ML models.

**Results:**

The performance of a random forest algorithm was high (area under the curve [AUC] = 0.89). As expected, surveillance-dependent variables for adjacency to LD-endemic counties (gain ratio: 0.22) and presence of *I. scapularis* (gain ratio: 0.133) were identified as the top individual predictors of LD spread. However, the strongest overall contributions to the model were driven by vertebrate-related variables (*n* = 8) (ReliefF: 0.237), with landscape features for forest growth, canopy, and forest edge (length) also identified as strong (gain ratio > 0.28) individual predictors. Climate predictors indicated the lowest LD incidence classes (< 10 cases/100,000 population) in warmer and drier counties and the highest LD incidence classes (> 10 cases/100,000 population) in the coldest and wettest counties.

**Conclusions:**

Utilization of ML algorithms trained with variables impacting the circulation of *Bb* produced a comprehensive model of county-level LD incidence and captured the main factors acting on the spread of the pathogen. This represents an important step towards an integrated framework aimed at capturing LD incidence changes for preventive purposes.

**Graphical Abstract:**

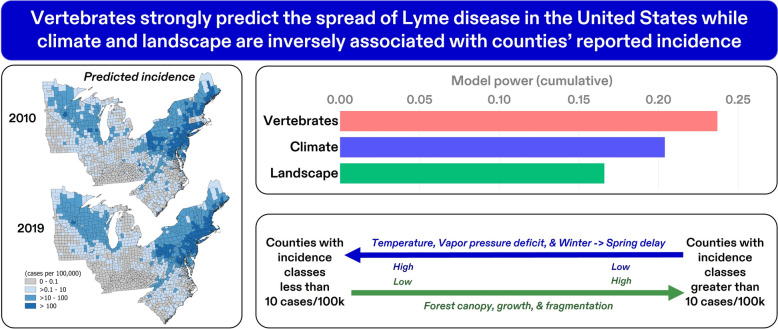

**Supplementary Information:**

The online version contains supplementary material available at 10.1186/s13071-025-07047-9.

## Background

Lyme disease (LD) is the most widely reported vector-borne disease in North America, with estimates of ~476,000 cases treated annually in the United States [1–3]. Over the past three decades, the incidence of LD has rapidly increased in the USA, in part due to the geographical spread of the primary tick vector *Ixodes scapularis* infected with *Borrelia burgdorferi* sensu lato (*Bb*), the bacterial complex of genospecies that cause LD [4]. In the USA, LD incidence is largely geographically restricted to the northeast, mid-Atlantic, and Midwest regions, where 16 jurisdictions (15 states and Washington D.C.) are considered high incidence for LD (reporting ≥ 10 cases/100,000 population over three consecutive years) by the Centers for Disease Control and Prevention (CDC) as of 2022 [5]. Based on the historical trends and most recent data reported to the CDC, the number of high-incidence LD states and counties in the USA is expected to rise [6].

The natural transmission cycle of LD involves small mammals, particularly the white-footed mouse (*Peromyscus leucopus*), which serves as a highly competent reservoir host of *Bb* [7]. Immature ticks acquire *Bb* by feeding on infected reservoirs and may transmit it to naïve hosts during subsequent life stages, especially as nymphs, which are the primary vectors to humans due to their small size and peak activity in late spring and early summer [8]. White-tailed deer (*Odocoileus virginianus*) play a key role as reproductive tick hosts to increase and maintain tick populations, although they are not competent reservoirs for *Bb* [9]. The distribution and abundance of *I. scapularis* are strongly influenced by climate variables such as temperature and humidity [10], as well as landscape structure and forest fragmentation [11] and host availability [12]. Warmer winters and earlier springs have facilitated tick range expansion into northern latitudes and higher elevations [13]. Habitat features that support dense populations of reservoir hosts, such as deciduous forests with high understory vegetation, also increase LD risk. Consequently, these factors have all contributed to spatial spread and increase of reported cases in humans in the United States since the 1990s [14–18].

The likelihood of *Bb* transmission from infected *I. scapularis* ticks to humans is determined by a complex interplay between local environmental variables including climate, habitat type, and abundance of key vertebrates that impact the life cycle dynamics of *I. scapularis* and transmission rates between ticks and reservoirs, in relation to individual behaviors and outdoor activities that lead to exposure to *Bb*-infected ticks [19, 20]. Decoupling the multifactorial associations between these factors and the spatial dynamics of *I. scapularis* and LD incidence remains a challenge, mainly because of the need to incorporate the best explanatory variables that describe the epidemiological situation over a large territory (e.g., continent). There is a risk of inclusion of too many variables that are only regionally significant and tend to inflate the overall model across broader territories or to include descriptive variables that are statistically robust but are ecologically redundant or lack relevance (e.g., certain weather variables). Previous models of LD in the USA have attempted to identify and measure the environmental associations with reported incidence across different states and regions with varying outcomes [21–23]. Part of the reason for varying outcomes is due to limited surveillance data and spatial heterogeneity in the density of *Bb*-infected host-seeking *I. scapularis* nymphs, as documented in other modeling exercises [24–27]. Varying inclusion of explanatory variables as predictors of LD incidence also results in differential model outcomes. For instance, although it is well known that the circulation of *Bb* is dependent on key vertebrate reservoir species, and the reproduction of *I. scapularis* is driven by large mammalian hosts, animal-related explanatory variables are often omitted or unavailable from spatial analyses or restricted to small regions [16, 28, 29].

Machine-learning (ML) algorithms are suitable tools for complex spatial model development, classically trained with a set of known locations across a defined territory, that identify non-linear associations with explanatory variable features which are then applied across the remaining locations or territory. However, developing robust ML models to ascertain the ecology of LD and factors leading to high/low incidence across broad US territories remains challenging [30, 31]. Quantifying spatio-temporal risks of LD in the USA is further hampered by changing LD surveillance case definitions, resulting in varying reported incidence over time within and between states and counties, which can increase model bias and diminish reliability [17].

This study presents a comprehensive spatial ML model to predict LD incidence at the US county level. The model is based upon variables of the counties’ observed presence/absence of *I. scapularis* and their adjacency with high-incidence LD counties according to epidemiological surveillance data from the CDC, as well as important environmental features of LD for climate, vegetation, habitat landscape features, and most reported vertebrates that may be competent reservoirs for *Bb* or hosts for *I. scapularis.* Our aim is to better disentangle the ecological factors behind the observed spatial spread of LD in the USA over a 10-year period by developing a single model accounting for the LD cases reported per county and year in relation to changes in critical variables as an assessment of LD risk.

## Methods

### Lyme disease cases at the county level

Surveillance data for annual reported cases of LD per county per year (2010–2019) were obtained from the CDC [32]. Sixteen “high-incidence” jurisdictions, as defined by the CDC (15 states and Washington D.C.), and seven “low-incidence” states neighboring high-incidence jurisdictions (Iowa, Illinois, Michigan, Indiana, Ohio, Kentucky, and North Carolina, the “target territory”) were included in the model. Model training began for the year 2010 using the Council of State and Territorial Epidemiologists (CSTE)/CDC LD case definition updated in 2007 to include both confirmed cases (erythema migrans with known exposure to tick habitats or laboratory evidence of infection, or a case with at least one late-stage clinical manifestation and laboratory evidence of infection) and probable cases (clinically diagnosed cases with laboratory evidence of infection). Minor changes in the CSTE/CDC LD case definitions occurred in 2011 and 2017 [5]. Therefore, the training dataset included LD cases using case definitions established between 2007 and 2019. Data for the years 2020–2021 were omitted due to possible underreporting of LD associated with the COVID-19 pandemic.

To synthesize and restrict the modeled dependent variable of LD incidence to categories with epidemiological significance, we created four incidence classes for every county and year: (1) counties with zero or one annual case per 100,000 population reported were grouped to accommodate the negative counties and those with anecdotical reporting (cases are recorded as per county of residence, not the site of exposure); (2) counties that reported > 1 to 10 cases/100,000 population to accommodate counties below the high-incidence threshold defined by the CDC (mean cases > 10 cases/100,000 over a minimum 3 years of surveillance); (3) counties with > 10 to 100 cases/100,000 population; and (4) counties reporting > 100 cases/100,000 population. These classes are supported by preliminary examination of the ranges and significant differences among the model variables.

### Variables for modeling

Variables to predict the spatial spread of LD among US counties included features associated with the epidemiological surveillance data for LD and *I. scapularis*, climate, landscape, and vegetation, and expected distributions of vertebrate *I. scapularis* hosts and *Bb* reservoirs (Datafile S1). While climate is routinely used in the modeling of ticks, accounting for the *Bb* pathogen and tick presence needs to incorporate the presence/absence of their main hosts and reservoirs and landscape patterns that influence transmission.

Two epidemiological surveillance variables associated with the establishment of *I. scapularis* and reported LD incidence in the counties were derived and included for model training. The variable “county adjacency” for the counties adjacent to high-incidence LD counties was included in the model to explain the diffusion and spread of LD incidence across the “target territory” (i.e., low-incidence US states/counties) as proposed previously [33]. For each year in the time series of county case numbers, the high-incidence counties were identified, followed by the selection of their adjacent low-incidence neighboring counties. The latter selected counties were marked as “adjacent” for the next year in the series of data. The process was repeated year after year, selecting counties adjacent to those reporting high-incidence LD surveillance data.

The variable for counties’ annual reported *I. scapularis* presence was derived using tick surveillance data collected via tick dragging or flagging in the USA from available published reports [34–36] and complemented with data from VectorMap [37] and the Global Biodiversity Information Facility (GBIF) [38]. Counties were classified as “established” for *I. scapularis* when at least one of the resources provided verified information about the presence of reproducing populations, and the remaining counties were classified as “absent,” since we could not adhere to the defined category scheme of “absent,” “reported,” and “established,” because that information was unavailable over the complete time series in the sources used [39, 40]. To account for annual gaps in the *I. scapularis* dataset, climate variables were included as probable drivers of the annual geographical range of *I. scapularis* over time for the entire target region, as climate is known to affect the spread of ticks [10, 15, 41, 42]. Changes to counties’ *I. scapularis* presence status were evaluated for every year using a stacked species distribution modeling approach [43–45]. The model was trained with the data regarding the distribution of *I. scapularis* in the year 2019 and projected backward with the climate variables of each year; these annual raster data were transferred to the counties. A county was considered positive for *I. scapularis* when the ensemble modeling outcome was higher than 50% for a county, because this threshold produced the best result for the year 2020, with 91% accurately classified counties (data not shown).

Climate data associated with counties’ temperature and water vapor pressure deficit (VPD) were obtained between 2010 and 2019 from the TerraClimate repository [46]. County-level data were summarized as the monthly average for each individual year followed by three coefficient derivations of harmonic regression for incorporation as explanatory variables (*n* = 9) [47]. The first coefficient of climate variables was the annual average VPD, average maximum temperature, and average minimum temperature; the second coefficient variables were derived to explain the counties’ seasonal “sharpness” of temperature and VPD from winter to spring (termed “spring slope”). A cold winter and a warm spring result in a sharp spring rise (increased values), while a mild winter produces a blunt spring rise (decreased values). A third coefficient is always available in harmonic regression that would explain the counties’ seasonal sharpness of temperature and VPD deficit from summer to autumn (termed “autumn slope”), though none were significant during model development and were excluded from the final model.

The importance of landscape features in the ecology of LD has been pointed out in field studies but rarely addressed in models for a large region [11, 27]. Variables for habitat features were obtained from the United States Geological Survey (USGS) in the years 2010, 2013, 2016, and 2019 at a resolution of 100 m [48]. The variables included were (i) an estimate for forest fragmentation forest edge length (median); (ii) woodland proportion (%) of forest growth type (deciduous, coniferous, mixed), (iii) the proportion (%) of forest canopy; and (iv) accessibility, defined as the distance between human settlement(s) and vegetated areas to quantify the proximity or potential contact rate between wild animals and humans (higher values indicate closer proximity between animals and humans). The habitat variables for forest edge length, forest growth type, forest canopy, and accessibility were calculated as the sum of the pixels in the original data for each county, divided by the area of such county. All the spatial statistics were obtained using qGIS software [49]. Satellite-derived images were obtained from the MODIS [Moderate Resolution Imaging Spectroradiometer] website of the National Aeronautics and Space Administration (NASA) using the package “MODIStsp” [50] for R, for the period 2009–2019. Variables for the normalized difference vegetation index (NDVI), a standard value of vegetation "greening,” were also included as habitat explanatory predictors of the spatial spread of LD accounting for the counties’ annual sum of NDVI in the current (“current year”) and preceding (“previous year”) years by summing the NDVI values of each 32-day period (as delivered by the MODIS team), after removal of pixels contaminated by clouds, ice, or snow by methods recommended by the MODIS research team and available at the MODIS website.

Data on the distribution of competent and non-competent *Bb* animal reservoirs or *I. scapularis* hosts were obtained from high-resolution maps provided by the USGS Gap Analysis Project [51] to calculate the geographical range of competent reservoirs for *Bb* in the target territory. The rationale for selecting species of vertebrates is based on the known fact that some vertebrates may impact *Bb* circulation [52]. Vertebrates involved in the epidemiology of *Bb* and/or tick hosts were selected based on the list of hosts provided in VectorMap and the available collections in the US National Tick Collection (provided by Dr. Lance Durden). Animal distributions at 100 m resolution were collected for the northern short-tailed shrew (*Blarina brevicauda*), white-footed mouse (*P. leucopus*), white-tailed deer (*O. virginianus*), bobcat (*Lynx rufus*), raccoon (*Procyon lotor*), gray fox (*Urocyon cinereoargenteus*), Virginia opossum (*Didelphis virginiana*), and broadhead skink (*Eumeces laticeps*). To incorporate these data into the modeling approach, the number of positive pixels in each county were summed and divided by the total area of the county to derive the percent of available habitat for each vertebrate in each county. These were static explanatory variables in the model for the whole study period because the maps on vertebrate distribution have been prepared, curated, and built by experts for the years 2010–2019, and have not been updated. These species were selected based on their importance as hosts of the tick vector (e.g., white-tailed deer, short-tailed shrew, Virginia opossum) or competent reservoirs of *B. burgdorferi* (e.g. white-footed mouse) or lack of importance as either hosts of the ticks or spreaders of the infection (e.g., bobcat, broadhead skink). While available in the USGS Gap Analysis Project [51], the selection of other vertebrates was not considered because of their distinct regional distributions, which may over-tune the overall model, and the lack of suitable data for birds that may behave as spreaders of ticks. In summary, we sought to have a balanced representation of vertebrate hosts with geographical distributions across the target territory and varying influence on the natural enzootic cycle of LD included for model development.

### Machine-learning algorithm development to model the annual cases of Lyme disease

Five modeling algorithms were used to predict the incidence of LD in counties in each year: (1) gradient boosting, (2) random forest (RF), (3) AdaBoost, (4) neural networks, and (5) naive Bayes. Gradient boosting is a regression and classification algorithm that produces a prediction model in the form of an ensemble of decision trees. For gradient boosting, we used 100 trees and a learning rate of 0.3, with lambda = 3. RF also builds decision trees. Each tree of decisions is trained on a random subset of data, and each split is based on a random subset of the features bootstrap sampling and feature bagging to reduce the overfitting of individual trees. For RF, we used 10 trees, with replicate training, without balancing the class distribution. The AdaBoost (adaptive boosting) is an algorithm that can be used with other learning algorithms to boost their performance. For AdaBoost, we used the option “tree” as base estimator, with 50 estimators and a learning rate of 1, using the SAMME.R as classification algorithm and the linear regression loss function. The neural networks used 100 neurons in one hidden layer, activated by the rectified linear unit (ReLu) function and the l-BFGS-*B* solver, with alpha = 0.0001 and 200 maximum iterations. For naive Bayes, we assumed equiprobable classes (i.e., priors = 1/[number of classes]).

Algorithms were trained with the complete series of reported LD incidence according to the defined categorized incidence classes to produce the model-predicted values of these categories, and selected the best model based on model performance. The chosen model was then applied to each year separately to capture the evolution of LD incidence classes over the years 2010, 2013, 2016, and 2019, followed by applying the best model over the complete time series (2010–2019) and then testing model reliability with the actual reported LD surveillance data. Tenfold cross-validation was used to generate performance statistics in each algorithm, including the receiver operating characteristic (ROC) curve and the resulting area under the curve (AUC). Other validation methods included classification accuracy (CA), the proportion to correctly classify the observed data; precision (Prec.), the proportion of true positives across all observations classified as positive, calculated as [true positive/true positive + false positive]; and recall, defined as the proportion of true positives among the positive data observations only expressed as [true positive/true positive + false negative]. Calculations to measure the importance of the explanatory variables were gain ratio (the predictive power of an individual variable to inform the model relative to its uncertainty) and ReliefF (an iterative heuristic for ranking variable importance in a model based on each variable’s ability to discriminate different target outcomes according to contribution to inform the overall model including all explanatory variables). Non-significant variables were dropped from models, and the circumstance explained in the Results section. The ML algorithms were developed using the Orange data mining environment [53]. The complete script for model development is provided (Datafile S2). A multivariate analysis of variance (MANOVA) was performed on the explanatory variables grouped by year, by the categorized LD incidence classes, or both factors together to discern the directional association(s) (positive or negative) of the explanatory variables for better interpretation of the ecological meaning of these results.

## Results

### Algorithm performance and selection of best model

**Fig. 1:**
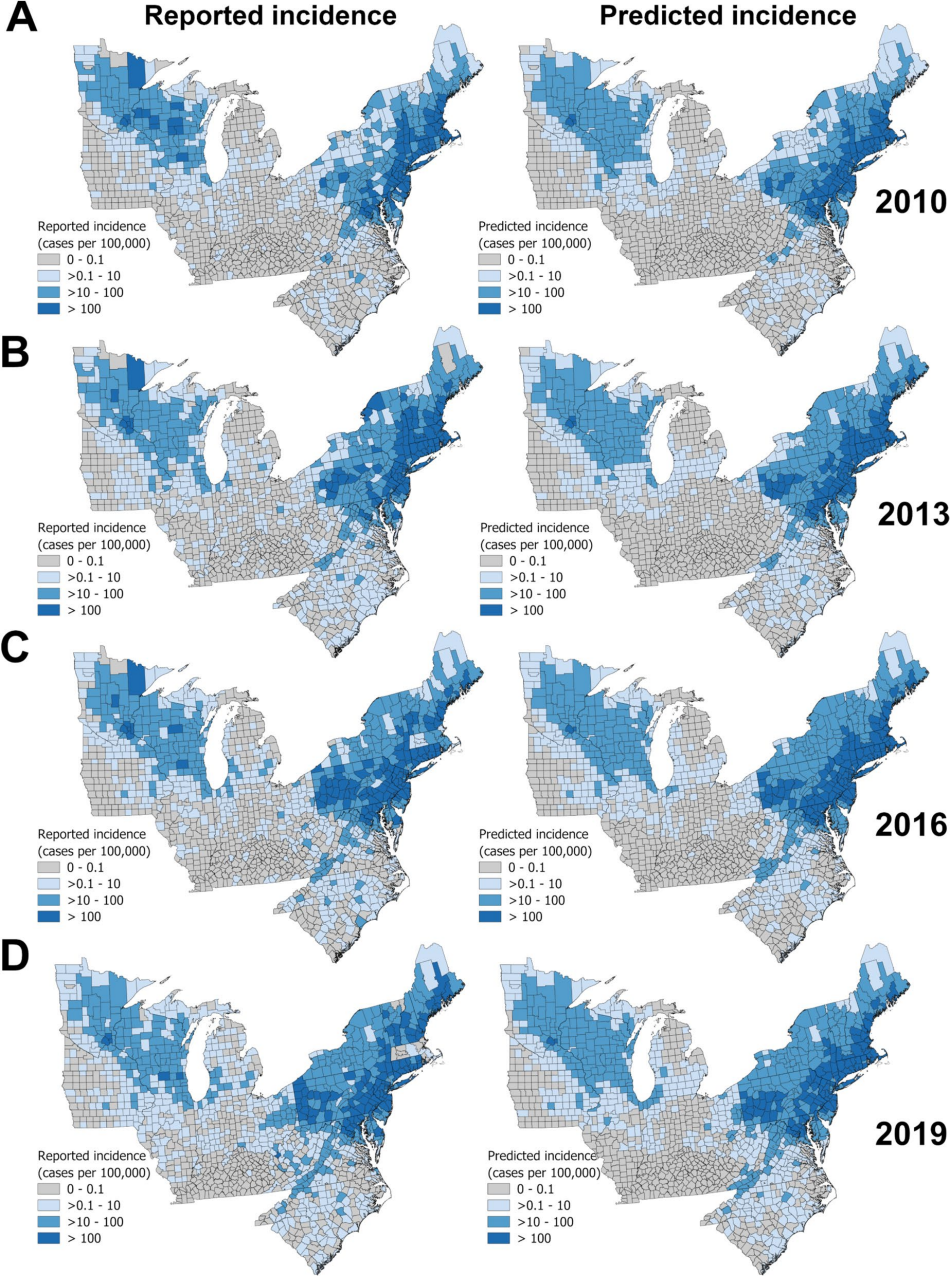
Comparison of annually observed versus predicted Lyme disease (LD) incidence in the UnitedStates, 2010-2019. Figures illustrate the reported LD incidence (left panels) or the developed randomforest machine-learning algorithm-predicted LD incidence (right panels) among 1322 US counties for theyears 2010 (**A**) , 2013 (**B**) , 2016 (**C**) , and 2019 (**D**) according to national surveillance data from theCenters for Disease Control and Prevention (CDC)

Five ML algorithms were tested for performance predicting LD incidence in 1322 counties of the USA, using indexes of model accuracy and reliability with various sets of eligible variables. Gradient boosting was slightly superior when trained and applied to several years jointly modeled (Table 1); however, we chose to use the RF algorithm based on its highest performance metrics when trained with the complete dataset over the entire time series and then applied for each individual year 2010, 2013, 2016, and 2019 compared to the gradient boosting approach (Table S1). The RF model outperformed the gradient boosting model when predicting LD incidence class for 1322 counties each year (Table S1). Outcomes from the RF model varied among years but correctly classified 79.8–94.6% of the counties across all incidence classes compared to the gradient boosting model, which was less accurate (range 61.0–90.8%) for all years and incidence classes (Table S1). Overall, the RF model accurately predicted 93.4% of the counties with a reported LD incidence class < 1/100,000 population, and 89.7% and 93.0% of counties that reported LD incidence of ≥ 10 or > 100/100,000 population, respectively (Table 2). The RF algorithm was less accurate overall (81.6%) for identifying counties with reported LD incidence between 1 and 10 cases/100,000 population (Table 2). A side-by-side comparison of the annual reported and RF model-predicted LD incidence according to incidence classes for the years 2010, 2013, 2016, and 2019 is shown in Fig. 1a, b, c, and d, respectively.

**Table 1:** Performance of machine-learning spatio-temporal algorithms to predict Lyme disease incidence in the United States, 2010–2019

Algorithm	AUC	CA	Prec.	Recall
Gradient boosting	0.907	0.748	0.743	0.748
Random forest	0.895	0.742	0.738	0.742
AdaBoost	0.759	0.953	0.638	0.681
Neural network	0.909	0.754	0.750	0.754
Naive Bayes	0.834	0.704	0.492	0.531

Algorithm performance was evaluated using the complete set of explanatory variables when choosing the best set of explanatory variables in the final algorithm model over the complete time series of reported cases in each county

**Table 2:** Confusion matrix of the random forest (RF) algorithm county classification of Lyme disease incidence classes

			Random forest algorithm-predicted incidence			
		0–1	> 1 to 10	> 10 to 100	> 100	∑
Observed incidence (2010–2019)	0–1	93.4%	6.2%	0.3%	0.1%	2202
	> 1 to 10	14.1%	81.6%	3.9%	0.4%	1628
	> 10 to 100	0.7%	7.1%	89.7%	2.5%	1053
	> 100	0.0%	0.2%	9.1%	90.6%	405
	∑	2292	1542	1051	403	5288

Table shows the proportion (%) of counties correctly classified (columns: RF-predicted) with their reported (rows: actual observed) Lyme disease incidence classes of 0–1 case, > 1 to 10 cases, > 10 to 100 cases, and > 100 cases per 100,000 population according to the United States Centers for Disease Control and Prevention for the period between 2010 and 2019

### Identification of ecological variables most associated with the spatial pattern of Lyme disease

**Fig. 2:**
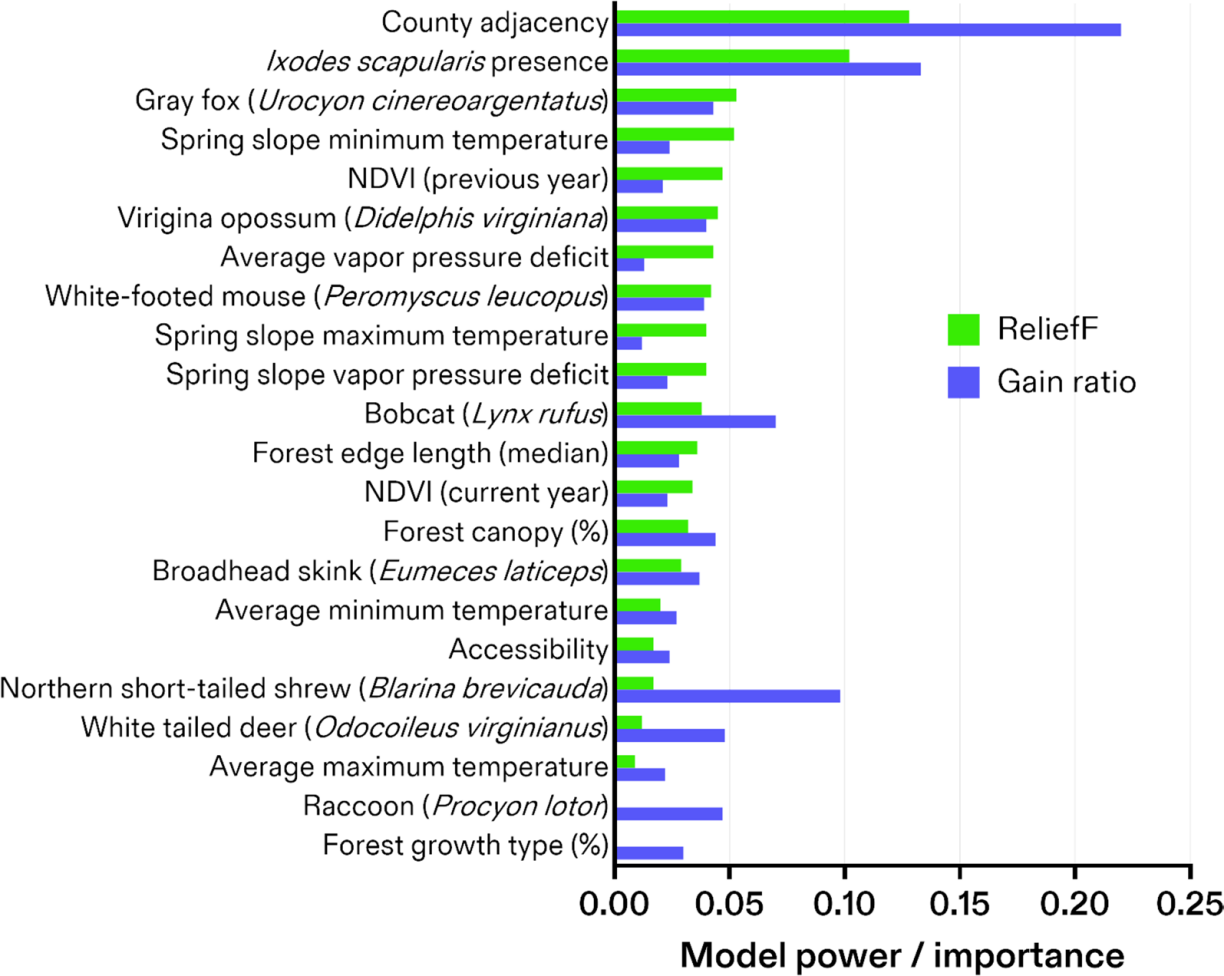
Explanatory variable ranking in a random forest machine-learning spatio-temporal algorithm of Lyme disease. Explanatory variables are sorted from top (strongest predictors) to bottom (lowest predictors) according to their net contributions to the overall model (green bars; ReliefF). Each variable’s individual power to predict spatio-temporal Lyme disease incidence is illustrated (blue bars, gain ratio). Variables with a gain ratio < 0.01 were excluded

**Fig. 3:**
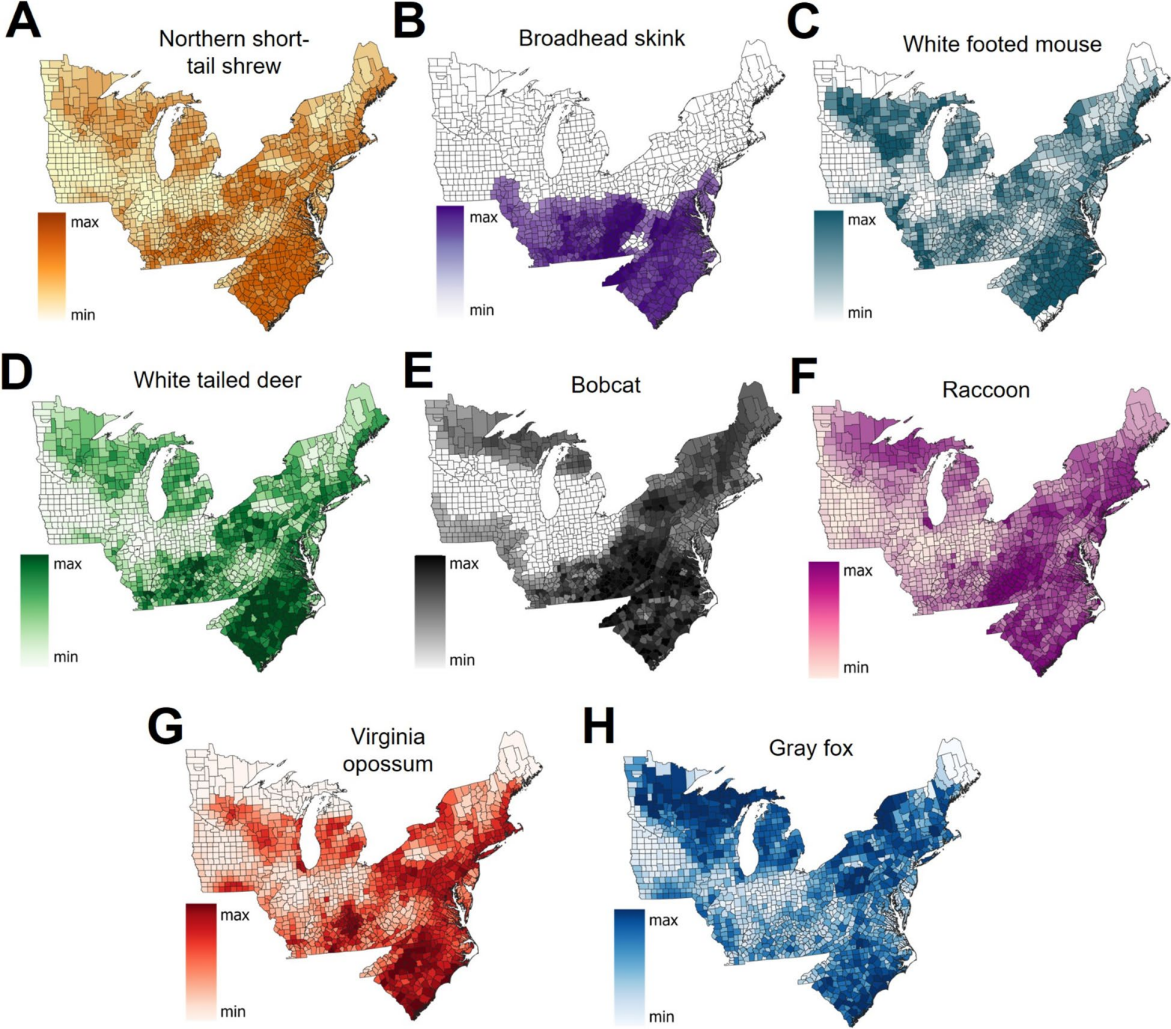
Expected geographical distribution of vertebrate host variables included for spatio-temporal modeling of Lyme disease incidence. Relative habitat suitability data were estimated for the geographical ranges of the following species and included for the development of a random forest machine-learning algorithm of Lyme disease in the United States, 2010–2019: **A** northern short-tailed shrew (*Blarina brevicauda*); **B** broadhead skink (*Eumeces laticeps*); **C** white-footed mouse (*Peromyscus leucopus*); **D** white-tailed deer (*Odocoileus virginianus*); **E** bobcat (*Lynx rufus*); **F** raccoon (*Procyon lotor*); **G** Virginia opossum (*Didelphis virginiana*); **H** gray fox (*Urocyon cinereoargenteus*). The legend indicates the number of suitable pixels of 100 m resolution (as provided by the USGS Gap analysis Project) in each county

Several variables were identified with high predictive power and importance in building the best model (Fig. 2). The strongest (gain ratio) variables to explain spatial diffusion of LD in the model were the surveillance variables for county adjacency (number of years in which a county was adjacent to other high-incidence LD counties) (0.220) followed by the counties with observed *I. scapularis* presence (0.133). The next best set of explanatory variables were related to the expected distributions and suitability for several vertebrate species (Fig. 3). The northern short-tailed shrew (0.098) had the highest predictive power out of vertebrate-related variables, followed by the bobcat (0.070), white-tailed deer (0.048), and raccoon (0.044) (Fig. 2). Aggregate model importance (ReliefF) from all vertebrate host variables was higher (0.237) than any other category of explanatory variables but was followed closely by the aggregate from the surveillance variables of county adjacency (0.128) and *I. scapularis* presence (0.102) (Fig. 2). Notably, despite having lower predictive power than the vertebrate species, the gray fox (third), Virginia opossum (sixth), and white-footed mouse (eighth) were the highest-ranked vertebrate-related variables and provided the majority (59%) of the vertebrate-related contributions to the overall model.

The next best explanatory predictors were the landscape variables for forest canopy (0.044), forest growth type (0.030), and forest edge length (0.028) (Fig. 2). The final model determined that variables for climate were generally the weakest explanatory predictors (< 0.027 for all variables). However, their contribution to the overall model (ReliefF) ranked higher. This list of best explanatory variables drives the interpretation of the model outcome: the incidence of LD in counties is explained by a background of presence/absence of *I. scapularis* and their vertebrate hosts, over which landscape- and weather-derived variables influence temporal changes in reported incidence.

A MANOVA detected significant differences among the explanatory variables important for model development with the categories of LD incidence classes per county (Table 3). Significant differences were also identified for some of the climate and NDVI variables across the time series, but no consistent trend (e.g., increasing or decreasing) was observed. The variables included in the table were associated with significant differences among the incidence classes; moreover, climate variables had differences among both the years and incidence classes.

**Table 3:** Multivariate analysis of variance results for explanatory variable associations with Lyme disease incidence classes

Explanatory variable	Source term	*df*	Sum of squares	Mean square	*F*-ratio	*P*-value	Power *α* = 0.05
Epidemiology surveillance							
County adjacency	Incidence classes	3	264,063	88,021	5995	< 0.001*	1
Landscape; vegetation							
Accessibility (median)	Incidence classes	3	356.4	118.8	392.6	< 0.001*	1
Forest type (median %)	Incidence classes	3	2.81E+07	9.37E+06	126.6	< 0.001*	1
Forest canopy (median %)	Incidence classes	3	418,466	139,489	99.6	< 0.001*	1
Forest edge length (median)	Incidence classes	3	2121	707.1	83.9	< 0.001*	1
Average NDVI (previous year)	Incidence classes	3	135.5	45.2	33.7	0.009*	1
	Years × Incidence classes	30	68.2	2.3	1.7	< 0.001*	0.996
Average NDVI (current year)	Incidence classes	3	131.3	43.8	31.8	< 0.001*	1
	Years × Incidence classes	30	71.9	2.4	1.7	0.007*	0.997
Vertebrate hosts							
*Eumeces laticeps*	Incidence classes	3	1.41E+16	4.69E+15	735.8	< 0.001*	1
*Urocyon cinereoargenteus*	Incidence classes	3	4.00E+14	1.33E+14	537.6	< 0.001*	1
*Procyon lotor*	Incidence classes	3	6.65E+15	2.22E+15	276.8	< 0.001*	1
*Didelphis virginiana*	Incidence classes	3	3.13E+15	1.04E+15	146.2	< 0.001*	1
*Odocoileus virginianus*	Incidence classes	3	3.15E+14	1.05E+14	70.4	< 0.001*	1
*Peromyscus leucopus*	Incidence classes	3	1.24E+15	4.12E+14	66.3	< 0.001*	1
*Blarina brevicauda*	Incidence classes	3	1.18E+15	3.95E+14	50.7	< 0.001*	1
*Lynx rufus*	Incidence classes	3	1.80E+15	6.00E+14	38	< 0.001*	1
Climate							
Average maximum temperature	Incidence classes	3	17,743	5914	660.7	< 0.001*	1
	Years × Incidence classes	30	2440	81.3	9.1	< 0.001*	1
Average minimum temperature	Incidence classes	3	15,983	5327	617.9	< 0.001*	1
	Years × Incidence classes	30	442.4	14.7	1.7	0.009*	0.996
Spring slope vapor pressure deficit	Incidence classes	3	3.2	1.1	248.5	< 0.001*	1
	Years × Incidence classes	30	1.3	0.0	10.1	< 0.001*	1
Spring slope maximum temperature	Incidence classes	3	3228	1076	238.5	< 0.001*	1
	Years × Incidence classes	30	1185	39.5	8.8	< 0.001*	1
Spring slope minimum temperature	Incidence classes	3	1086	361.9	172.8	< 0.001*	1
	Years × Incidence classes	30	121.4	4.0	1.9	0.002*	0.999
Average vapor pressure deficit	Incidence classes	3	481.6	160.5	4.2	0.006*	0.857
	Years × Incidence classes	30	3263	108.8	2.8	< 0.001*	1

The analysis was carried out over each year of the time series (2010–2019), the categories of annually reported Lyme disease (incidence classes; 0–1 case, > 1 to 10 cases, > 10 to 100 cases, and > 100 cases per 100,000 population), and their product (year × incidence class). Included are the degrees of freedom (*df*), the vales of the mean square, the *F*-ratio, and the probability level. A *P*-value < 0.05 was considered statistically significant

**Fig. 4:**
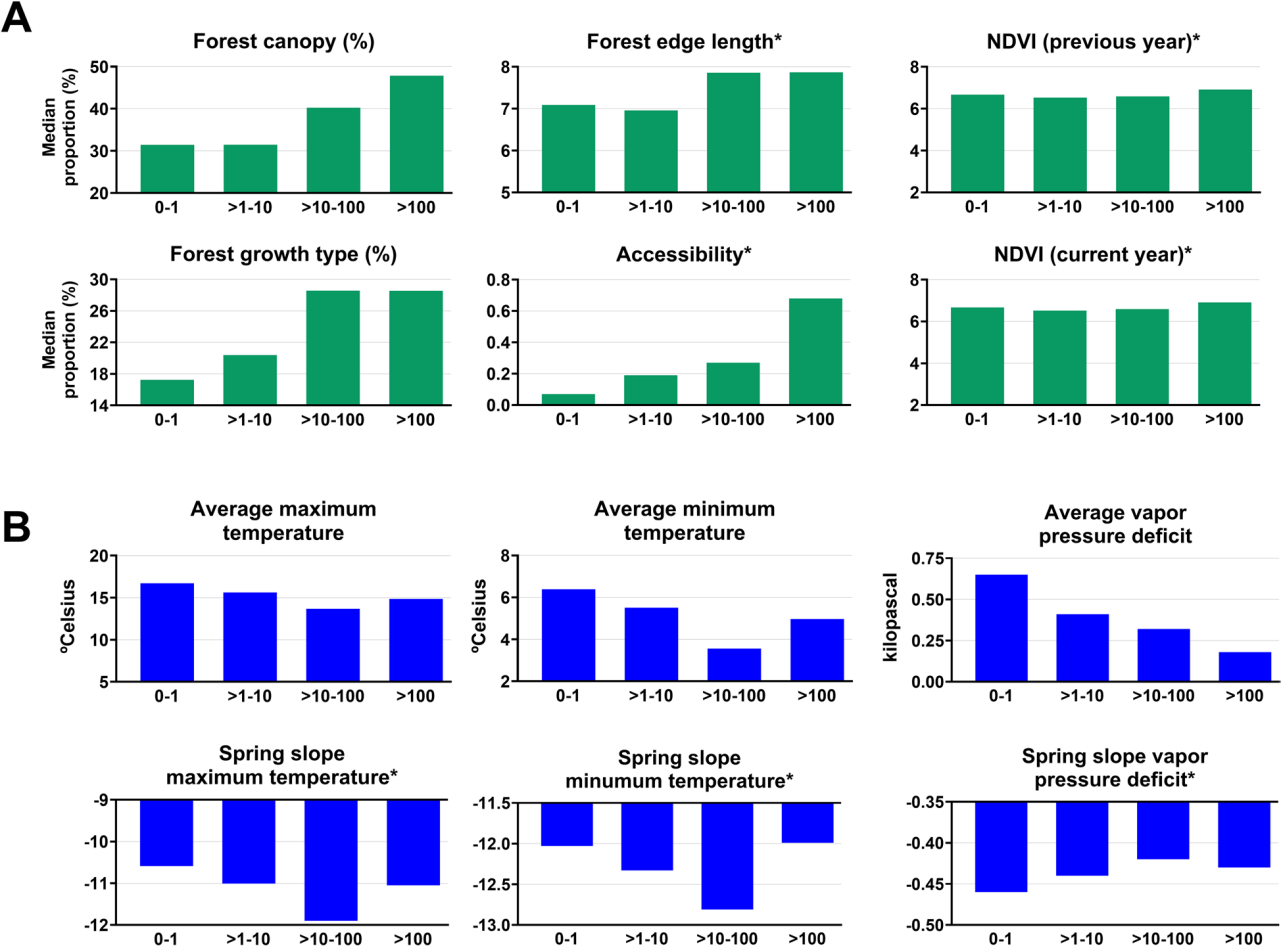
Directional associations between environmental variables and Lyme disease incidence classes among US counties. Results were determined according to a multivariate analysis of variance (MANOVA) between abiotic explanatory variables for landscape and vegetation (**A**) or climate (**B**) and US counties’ annual reported Lyme disease incidence classes of 0–1 case, > 1 to 10 cases, > 10 to 100 cases, and > 100 cases per 100,000 population between 2010 and 2019. Graph titles with an asterisk (*) denote explanatory variable values that are unit-less, with N/A for the *y*-axis

Next we investigated the associations between the environmental predictors and the counties’ LD incidence classes. Counties in the upper (> 10 cases/100,000 population) LD incidence classes are associated with increased forest canopy, forest growth type, forest edge length, and animal accessibility (Fig. 4a). Regarding the weather variables, counties reporting 0–1 case/100,000 population are the warmest (higher average maximum temperature) and driest (higher average VPD), while counties in higher (> 10 cases/100,000 population) LD incidence classes are associated with colder (lower average minimum temperature) and drier (lower average VPD) climates (Fig. 4b). Neither NDVI variable for accumulated NDVI in the current or previous year showed differences across the incidence classes (Fig. 4a), and they were considered lower-ranked variables for model development (Fig. 3). Differences were observed in the values of the spring slope of maximum and minimum temperature (Fig. 4). Counties reporting 0–1 or > 1 to 10 cases/100,000 population have a more gradual increase in spring temperatures than counties that report > 10 to 100 cases/100,000, which have a faster, sharp transition to higher spring temperatures. However, counties in the top incidence class reporting > 100 cases/100,000 population have the highest spring slope of minimum temperature values among all incidence classes, indicating that these counties have a slight transition of minimum temperature from winter to spring. The reported values associated with each explanatory variable and LD incidence class are provided in the additional files (Table S2).

## Discussion

An ML algorithm trained with a broad set of epidemiologically relevant variables as explanatory predictors of LD is highly suited to track county-level incidence of LD across the USA. Including human and tick surveillance data with variables related to climate, landscape, vegetation, vertebrate tick hosts, and *Bb* reservoir allowed the RF model to identify counties with annual reported LD incidence of < 1 to > 100 cases per 100,000 population with high sensitivity and precision over time. Although previous models predicting LD incidence have been built, they used smaller sets of explanatory variables, limiting data interpretation and generalization [54]. Importantly, this approach used a 10-year series over a wide territory involving 1322 counties with varying LD endemicity, and demonstrated the utility of ML methods to accurately identify where LD spreads. Due to the complexity of the *Bb* enzootic cycle between *I. scapularis* and animals, coupled with the highly multifactorial abiotic components, rigorous modeling of LD should not be performed with a small number of explanatory variables [14, 27, 55, 56]. Models incorporating insufficient variables may partly explain the spatial distribution of reported LD cases, but it is necessary for the *joint* inclusion of other explanatory variables for algorithm training to reach adequate sensitivity and specificity [57]. This model utilized relevant environmental predictors related to tick ecology and LD spread, resulting in increased reliability.

The incorporation of a variable accounting for county adjacency was critical to explaining the heterogeneous diffusion of reported LD cases over time. Similar variables for spatiality have been identified in other novel modeling approaches [17, 58]. Bisanzio et al. [17] found that counties with increased delays reporting their first LD case were more likely to have fewer neighboring adjacent counties with reported cases. Kugeler [58] developed a relative risk (RR) metric of LD using the observed versus expected number of LD cases adjusted for population differences across counties and time. The specificity and sensitivity of the model are particularly reinforced with the addition of that topological value, considering the particular diffusion features in the spread of the active foci [33].

It is interesting to note that the rough distribution of reported cases in the target territory largely follows the presence/absence patterns of some vertebrates included as explanatory variables. This seems to be due to clear regional disparities in the feeding availability and host-seeking behavior of *I. scapularis* for different animal host and reservoir species across the region [59]. Notably, the model identified strong associations between higher incidence classes and both competent (short-tailed shrew) and non-competent (broadhead skink) reservoirs with varying distributions across the USA. Despite our modeling approach being unable to determine whether an individual variable is a positive versus negative driver of spatial spreading of LD, we can infer that the broad distribution of the competent reservoir short-tail shrew has a positive impact on LD incidence (e.g., more spreading), while the geographically restricted distribution to the southernmost region of the incompetent reservoir broadhead skink has a negative impact on LD incidence (e.g., less spreading). As previously reported, the prevalence of *Bb*-infected ticks may be related to the number of encounters with competent *Bb* reservoir hosts like the short-tail shrew or white-footed mouse [60]. Our approach included estimated host distributions (of both competent and incompetent species) as a proxy for the abundance of vertebrates and a categorical classification of tick presence to reflect tick–reservoir encounter frequencies, which was effective for modeling LD incidence classes and risk over time. As noted, it is necessary to further explore the importance of these large-scale vertebrate distribution patterns with respect to the epidemiological trends of LD in the USA and to ensure these variables are incorporated when modeling tick-borne diseases for increased reliability [54].

It has been reported [11, 15, 61, 62] that the ecological traits promoting high exposure to *Bb* in the USA are derived from reforestation, growth of secondary and young forest, providing shelter for the reservoirs of the pathogen, as well as suitable climate that contribute to the survival of populations of the tick vector. This study found statistically significant variations on critical variables that govern the model. As a rule, counties reporting a higher incidence of LD have larger forested areas, more canopy, and a longer forest edge (e.g., increased forest fragmentation), and the animals observe closer contact with human structures. The importance of landscape and vegetation variables is recognized as a signature providing both abundant populations of vertebrate host and competent reservoirs, as well as the necessary microclimate for ticks [16, 56, 63]. Further, certain configurations of the habitat could support higher contact rates between ticks and reservoirs, supporting permanent foci of *Bb*, as already reported [64].

Climate variables were derived using a validated approach based on the coefficients of a temporal harmonic regression to define both their means and seasonal dynamics that outperform pre-tailored climate datasets [65–68]. It is well established that changing climate trends are one of the drivers behind geographical expansion of *I. scapularis* across North America contributing to the observed increase in LD cases [10, 69]. Every analysis on the climate variables included in this study determined unique climatic features in counties reporting incidence > 100 cases/100,000 population compared to counties reporting < 100 cases/100,000 population. We conclude that these different climate conditions, among other variables, may explain why some counties have a hyper-endemic LD status. Results pinpointed that in the spatial range targeted in this study, warmer and drier counties reported the smallest incidence (0–1 case/100,000 population in our incidence classes). Conversely, counties that have a slow spring temperature rise, a mean summer temperature range of 20–22 °C, and high humidity (low VPD) have high rates of LD > 10 cases/100,000 population. This is in line with contemporary studies of the climatic impact on *I. scapularis* survival. However, our findings are contrary to the hypothesis that an abrupt spring rise in temperature favors the synchronous seasonality of both ticks and reservoirs, subsequently increasing the likelihood of *Bb* infection [70, 71]. The interpretation of the climate values points to high-LD-rate counties as those with the best-performing tick life cycle, optimizing survival (low evaporation rates) and metabolic rates (temperature above a critical threshold). To note, hyper-endemic counties (reporting an incidence > 100 cases/100,000 population) are warmer than counties reporting incidence of > 10 to 100 cases/100,000 population, but VPD is lower, which supports higher tick survival [72, 73].

While MANOVA identified that climate values differed significantly among years, the short temporal period used in our study makes it impossible to identify changing trends. It is likely that the high-incidence LD counties in the USA have already reached a climate stability point. Burtis et al. (2016) studied the differences in weather variables among recently endemic regions (still showing an increase in LD incidence) and “stabilized” areas, which correspond to long-term endemic regions, reporting that their study only managed to detect reduced LD incidence in years with hot, dry summer weather within the stabilized areas because of the decrease in questing activity of *I. scapularis* [74]. We hypothesize that the active foci of *Bb* are still pervading into the LD region in the USA, an area that became climatically suitable for the reservoirs and the ticks an undetermined time ago.

This study has limitations. Some of the year-to-year variability in annual LD incidence within and between counties is due to passive surveillance of LD and changing case definitions. Our model did not include anthropic variables such as human behavior, demographics, knowledge, attitudes, practices, and perceptions of tick-borne diseases, which are important factors that influence the frequency or probability of exposure to tick populations. Although we incorporated comprehensive explanatory variables in our model beyond the scope of most other models, some variable datasets, such as the animal reservoirs, were not available across all years of the study period. We were unable to harmonize the inclusion of every landscape- and vegetation-derived feature, updated on an annual basis. While some features of vegetation could be reproduced from the National Land Cover Database (NLCD), the calculation of features like fragmentation and imperviousness were not updated annually like other variables included for modeling. In any case, the simultaneous use of suitable climate variables plus static (i.e., not updated) distributional patterns of key vertebrates and the joint effects derived from vegetation and landscape patterns are able to produce suitable congruence for reliable modeling, though future models could incorporate year-to-year changes in these variables for even further accuracy.

Lastly, this report encourages the update of any data, biotic or abiotic, that could be important for human health programs designed to combat vector-borne diseases [54]. As public health resources are limited and constrained due to the continued rise of zoonotic disease spillovers, other data collection systems leveraging One Health approaches and data types are urgently needed [75]. Analytical tools such as social media [76], Internet-based search trends [77], and data “dashboards" [78, 79] that provide broad use and free access to available data should be further developed and utilized when mapping tick-borne disease risk. Data on human behavior and movement patterns are lacking and rarely included in tick-borne disease models, despite the primary goal of risk-based modeling approaches focused on public health intervention strategies [28, 80]. Collaborative networks across government, academia, and industry are also urgently needed to more effectively implement public health interventions and policies that curb tick-borne diseases [81]. We look forward to the future development of ML models using longer temporal intervals of human LD surveillance data to make future predictions on the spatial spread of LD in the USA.

## Conclusions

This work demonstrates the importance of including underutilized predictor variables like the habitat for vertebrate tick hosts and *Bb* animal reservoirs in ML algorithms to explain spatial diffusion of LD in the USA. This novel approach harmonizing inconsistent LD incidence surveillance data over space and time highlights the critical importance for congruence between training and explanatory variable data for maximum reliability in predictive models. Importantly, this model accurately informs and can prepare states and counties for the likely increase in LD incidence over the next decade, providing additional clarity for future surveillance priorities by public health agencies.

## Supplementary Information

Additional file 1 (Additional file 1: Datafile S1. An Excel spreadsheet file including (a) a description of each explanatory variable, (b) the complete raw data used in the development of this study, (c) the population and case report of each county for the complete study period (obtained from the CDC and US Census Office). The columns of sheets b and c have a numeric indication of the year. Additional file 2: Datafile S2. A script including the development of the model in Python language, running under the umbrella of Orange data mining software (open-access software). To run this script, a basic knowledge of Python language is required. Additional file 3: Table S1. The confusion matrixes of the number of correctly or incorrectly allocated counties regarding the actual cases classes (rows) versus those predicted by the random forest and gradient boosting models (columns); results include the performance metric results for the individually modeled years 2010, 2013, 2016, and 2019. Values listed correspond to the proportion (%) of counties correctly identified by the models with the reported Lyme disease incidence class for each year. ∑ corresponds to the number of counties allocated for each incidence class. Additional file 4: Table S2. Tabular results for the associations between abiotic explanatory variables for landscape, vegetation, and climate with the 1322 US counties’ Lyme disease incidence classes in the complete 2010–2019 series.

## Data Availability

The datasets supporting the conclusions of this article are included within the article and its additional files or publicly available and cited appropriately in the references. The code used for this study is available as Additional Files.
