# Supplementary material for: Variables for habitat and vertebrate hosts of Ixodes scapularis are the best ecological predictors of the spatial spread of Lyme disease in the United States (2010–2019)
Source: Parasit Vectors. 2025 Oct 14;18:410. doi: 10.1186/s13071-025-07047-9 (PMC12522553; doi:10.1186/s13071-025-07047-9)
Supplement: Supplementary file 1 — Additional file 1 (Additional file 1: Datafile S1. An Excel spreadsheet file including (a) a description of each explanatory variable, (b) the complete raw data used in the development of this study, (c) the population and case report of each county for the complete study period (obtained from the CDC and US Census Office). The columns of sheets b and c have a numeric indication of the year. Additional file 2: Datafile S2. A script including the development of the model in Python language, running under the umbrella of Orange data mining software (open-access software). To run this script, a basic knowledge of Python language is required. Additional file 3: Table S1. The confusion matrixes of the number of correctly or incorrectly allocated counties regarding the actual cases classes (rows) versus those predicted by the random forest and gradient boosting models (columns); results include the performance metric results for the individually modeled years 2010, 2013, 2016, and 2019. Values listed correspond to the proportion (%) of counties correctly identified by the models with the reported Lyme disease incidence class for each year. ∑ corresponds to the number of counties allocated for each incidence class. Additional file 4: Table S2. Tabular results for the associations between abiotic explanatory variables for landscape, vegetation, and climate with the 1322 US counties’ Lyme disease incidence classes in the complete 2010–2019 series. [file 13071_2025_7047_MOESM1_ESM.zip › Supplemental_File_S4.docx]

**Supplemental File S4.** Tabular results for the associations between abiotic explanatory variables for landscape, vegetation, and climate with the 1322 United States counties’ Lyme disease (LD) incidence classes in the complete 2010-2019 series. Variables with denoted (*) indicate unit-less values specific to the individual variable. All values are the median results for each variable within the LD incidence class.

|  | **Lyme disease incidence classes (cases / 100,000 population)** | | | |
| --- | --- | --- | --- | --- |
| **Variable** | **0-1** | **>1-10** | **>10-100** | **>100** |
| Forest canopy (%) | 31.43 | 31.46 | 40.25 | 47.86 |
| *SD* | *0.000* | *0.000* | *0.001* | *0.001* |
| Forest growth type (%) | 17.25 | 20.40 | 28.57 | 28.55 |
| *SD* | *0.003* | *0.004* | *0.005* | *0.008* |
| Accessibility* | 0.07 | 0.19 | 0.27 | 0.68 |
| *SD* | *0.000* | *0.000* | *0.001* | *0.001* |
| Forest edge lenth* | 7.09 | 6.96 | 7.86 | 7.87 |
| *SD* | *0.000* | *0.000* | *0.001* | *0.001* |
| NDVI (previous year)* | 6.67 | 6.53 | 6.59 | 6.92 |
| *SD* | *0.013* | *0.016* | *0.021* | *0.033* |
| NDVI (current year)* | 6.67 | 6.52 | 6.59 | 6.91 |
| *SD* | *0.013* | *0.021* | *0.021* | *0.013* |
| Average maximum temperature (ºC) | 16.70 | 15.62 | 13.68 | 14.85 |
| *SD* | *0.000* | *0.000* | *0.001* | *0.001* |
| Spring slope maximum temperature* | -10.59 | -11.01 | -11.90 | -11.05 |
| *SD* | *0.000* | *0.000* | *0.001* | *0.001* |
| Average minimum temperature (ºC) | 6.39 | 5.51 | 3.56 | 4.97 |
| *SD* | *0.000* | *0.000* | *0.001* | *0.001* |
| Spring slope minimum temperature* | -12.03 | -12.33 | -12.81 | -11.99 |
| *SD* | *0.000* | *0.000* | *0.001* | *0.001* |
| Average vapor pressure deficit (kilopascal) | 0.65 | 0.41 | 0.32 | 0.18 |
| *SD* | *0.000* | *0.000* | *0.001* | *0.001* |
| Spring slope vapor pressure deficit* | -0.46 | -0.44 | -0.42 | -0.43 |
| *SD* | *0.000* | *0.000* | *0.001* | *0.001* |
